# Death-Associated Protein-1 Plays a Role in the Reproductive Development of *Nilaparvata lugens* and the Transovarial Transmission of Its Yeast-Like Symbiont

**DOI:** 10.3390/insects15060425

**Published:** 2024-06-05

**Authors:** Jian-Bin Yu, Xin Lv, Qian Liu, Jia-Yu Tu, Xiao-Ping Yu, Yi-Peng Xu

**Affiliations:** Zhejiang Provincial Key Laboratory of Biometrology and Inspection & Quarantine, China Jiliang University, Hangzhou 310018, China; y17857687285@163.com (J.-B.Y.); lx2481476963@163.com (X.L.); 17344135037@163.com (Q.L.); 18858530559@163.com (J.-Y.T.); yxp@cjlu.edu.cn (X.-P.Y.)

**Keywords:** *Nilaparvata lugens*, DAP-1, RNAi, yeast-like symbionts

## Abstract

**Simple Summary:**

*Nilaparvata lugens* is a monophagous insect that poses a significant threat to food security in Asia. At present, the control of *N. lugens* mainly depends on application of insecticides and cultivation of resistant rice varieties. However, *N. lugens* develops resistance quickly, which makes it difficult to control. Death-associated protein-1 (DAP-1) plays crucial roles in cell growth, migration, autophagy, and apoptosis in mammals. However, its function in insects remains unclear. In the present study, we cloned and identified *N. lugens* DAP-1 (NlDAP-1). NlDAP-1 was expressed during all developmental stages and in all tissues of *N. lugens*, particularly higher in the ovaries of female adults. RNAi of NlDAP-1 expression significantly inhibited its expression, leading to premature death, delayed ovarian development, and fewer offspring of *N. lugens*. Additionally, an immunofluorescence experiment showed that NlDAP-1 was highly expressed when yeast-like symbionts (YLSs) entered *N. lugens* oocytes, and inhibiting the expression of NlDAP-1 disturbed the process. These results indicate that NlDAP-1 plays a crucial role in the reproductive development of *N. lugens* and the transovarial transmission of its YLSs, making DAP-1 as a potential target for RNAi to control *N. lugens*.

**Abstract:**

Death-associated protein-1 (DAP-1) plays a crucial role in cell growth, migration, autophagy, and apoptosis in mammals. However, its function in insects remains unclear. In the present study, we cloned and identified *Nilaparvata lugens* DAP-1 (NlDAP-1). NlDAP-1 was expressed during all developmental stages and in all tissues of *N. lugens*, being particularly higher in the ovaries of female adults. RNAi with double-stranded NlDAP-1 RNA significantly inhibited the expression of NlDAP-1, leading to premature death (dying seven days earlier), delayed ovarian development, and fewer offspring (76.7% reduction in eggs with 77.4% reduction in egg hatching rate). Additionally, an immunofluorescence experiment showed that NlDAP-1 was highly expressed when yeast-like symbionts (YLSs) entered *N. lugens* oocytes, and inhibiting the expression of NlDAP-1 disturbed the process; the RNAi of NlDAP-1 caused a 34.9% reduction in the YLSs that entered oocytes. These results indicate that NlDAP-1 plays a crucial role in the reproductive development of *N. lugens* and the transovarial transmission of its YLSs.

## 1. Introduction

*Nilaparvata lugens* (Stål) (Hemiptera: Delphacidae) is a monophagous insect that poses a significant threat to food security in Asia [1]. *N. lugens* dries and wilts rice plants by draining rice sap and can also spread rice viruses, causing significant damage to rice plants [2,3,4,5]. At present, the control of *N. lugens* mainly includes cultivation management, biological control, physical control, and chemical control. Chemical control is the main control method for *N. lugens* because of its high efficiency in killing pests, but the extensive use of insecticides has enhanced the resistance of *N. lugens* to insecticides. Planting resistant rice varieties is another effective method by which to control *N. lugens*, but the virulence of *N. lugens* changes quickly, which is thought to be related to its yeast-like symbionts (YLSs) because YLSs play important roles in the growth, development, and reproduction of *N. lugens*. YLSs can synthesize and provide essential amino acids, sterols, and fatty acids for *N. lugens*, participate in the synthesis of lipids in *N. lugens*, and provide energy for vitamin synthesis [6,7,8]. Thus, *N. lugens* is difficult to control, so it is necessary to find new control strategies. For example, RNA interference (RNAi) is a useful substitute method for controlling *N. lugens* based on target genes that are essential for growth and development [4,9]. *N. lugens* possesses a highly RNAi-sensitive system. Successful RNAi through targeting *N. lugens* genes has led to strong and long-lasting phenotypic effects, increased lethality, delayed development, decreased egg hatching and offspring of the population, tissue/organ morphologic defects, and altered feeding behavior. This makes a valuable gene repertoire available for breeding new rice varieties with satisfactory resistance against *N. lugens* in the field [10].

Death-associated protein-1 (DAP-1) is a highly conserved protein that regulates mammalian cell growth, adhesion, and migration through external apoptotic pathways [11,12,13,14,15,16]. In addition, DAP-1 interacts with IFN-β promoter stimulator-1 and p21 to participate in innate immunity, the inflammatory response, and cell senescence [17,18]. This is similar to the function of death-associated protein-3 (DAP-3), which is a molecule with a significant role in the control of both apoptosis and anoikis, and which can affect cell growth and migration by regulating apoptosis. Moreover, the potential regulatory function of mitochondrial DAP-3 is involved in cellular senescence [19,20]. However, the function of DAP-1 and DAP-3 in insects remains unclear. In our previous study, DAP-1 in *N. lugens* interacted with YLS enolase, which plays a crucial role in the attachment of the YLS to the ovariole epithelial plug during transovarial transmission in *N. lugens* [21]. This result suggests that DAP-1 may regulate the transovarial transmission of YLSs. Therefore, exploring the role of DAP-1 in *N. lugens* may provide a new target site for control. In the present study, we cloned *N. lugens* DAP-1 (NlDAP-1), performed a bioinformatics analysis, examined the NlDAP-1 expression patterns, and investigated its function using RNAi.

## 2. Materials and Methods

### 2.1. Insect Rearing

The *N. lugens* used in this study were originally collected from Yuyao (Ningbo, Zhejiang province, China) and maintained in the Climatron at China Jiliang University, Hangzhou, China. The insects were reared at 27 ± 1 °C, with a 16 h–8 h (light–dark) photoperiod, on the seedlings of rice variety “Taichung Native 1”, which is a susceptible rice variety without a resistance gene.

### 2.2. Cloning NlDAP-1 cDNA

Total RNA was extracted from *N. lugens* adult female samples with the MiniBEST Universal RNA Extraction Kit (TaKaRa, Dalian, China) according to the manufacturer’s instructions. The specificity of the RNA bands was confirmed by 1% agarose gel electrophoresis, and the concentration was measured using the NanoDrop 2000 spectrophotometer (Thermo Scientific, Waltham, MA, USA). Subsequently, 1 μg of total RNA was used for cDNA synthesis with the PrimeScript^TM^ II 1st Strand cDNA Synthesis Kit (Takara). To clone the NlDAP-1 sequence, a polymerase chain reaction (PCR) primer pair for NlDAP-1 was designed with Primer Premier 5.0 (Table 1), and the NlDAP-1 sequence was amplified using Premix Taq^TM^ (Takara). Following amplification, the PCR product was cloned into the pMD19-T vector (Takara) and sequenced.

### 2.3. Sequence Comparison and Phylogenetic Analysis

The NlDAP-1 protein sequence was compared with other DAP-1 sequences from other animals (Vertebrate, Lepidoptera, Diptera, Coleoptera, Hemiptera, Hymenoptera, Blattodea) available in the NCBI GenBank via blastP (https://blast.ncbi.nlm.nih.gov/Blast.cgi, accessed on 3 February 2024), and a phylogenetic tree was constructed using MEGA X (Mega Limited, Auckland, New Zealand) and the neighbor-joining method (bootstrap = 1000). Additionally, the isoelectric point, molecular weight, and signal peptide cleavage sites were predicted individually using ExPASy (https://web.expasy.org/compute_pi/, accessed on 3rd February 2024) and SignalP (http://www.cbs.dtu.dk/services/SignalP/, accessed on 3 February 2024). The conserved amino acid domains and phosphorylation sites were predicted and analyzed using CDD (https://www.ncbi.nlm.nih.gov/Structure/cdd/wrpsb.cgi, accessed on 3 February 2024) and CBS (http://www.cbs.dtu.dk/services/, accessed on 3 February 2024).

### 2.4. Real-Time Quantitative PCR Analysis

The NlDAP-1 mRNA level was analyzed via real-time quantitative PCR (qPCR). Total RNA was extracted from *N. lugens* using the MiniBEST Universal RNA Extraction Kit (Takara). RNA (1 µg) was used for reverse transcription in a 20 µL reaction volume with the Perfect Real-Time PrimeScript^TM^ RT reagent Kit and the gDNA Eraser (Takara). A specific qPCR primer pair was designed with Primer Premier 5.0, and *N. lugens 18S rRNA* (*Nl18S*) was used as the internal control (Table 1). qPCR was performed in triplicate on Step One Plus (ABI, Foster City, CA, USA) using TB Green^®^ Primix Ex Taq^TM^ II (Tli RNaseH Plus) (Takara). The qPCR program was 94 °C for 30 s, 40 cycles of 94 °C for 5 s, and 60 °C for 30 s. Three replicates were performed for each treatment. Based on the cycle threshold and logarithm of the copy concentration, the amplification efficiency of the NlDAP-1 and *Nl18S* primers was calculated, and they were both 105%. Variation in NlDAP-1 expression was evaluated using the 2^−∆∆Ct^ method [22].

To analyze the developmental expression pattern of NlDAP-1, total RNA was extracted from 1st to 5th nymphs, 1–7 day-old macropterous female adults, 1–7 day-old brachypterous female adults, 1–2 day-old macropterous male adults, and 1–2 day-old brachypterous male adults. To analyze the tissue-specific expression pattern of NlDAP-1, total RNA was extracted from the head, ovaries, gut, thorax, and fat bodies of macropterous females. To analyze the expression change of NlDAP-1 after dsRNA injection, total RNA was extracted from *N. lugens* at two, three, and five days post-dsRNA injection.

### 2.5. Immunofluorescence

Immunofluorescence analysis was performed to determine the localization and distribution of the NlDAP-1 protein. The ovaries were dissected from *N. lugens* and fixed and stained according to previously described methods [23,24]. The primary antibody (anti-DAP-1) was a mouse monoclonal antibody against the human DAP-polypeptide 1 (Santa Cruz Biotechnology, Santa Cruz, CA, USA) that had 76% similarity with NlDAP-1. The specificity of the primary antibody was verified via Western blot (Figure S1). The secondary antibody was a goat anti-mouse IgG antibody conjugated with DyLight 594 fluorescent dye (Abbkine, Santa Ana, CA, USA). The samples were observed with a laser scanning confocal microscope (Leica SP8, Mannheim, Germany).

### 2.6. RNA Interference

A primer pair (dsNlDAP-1-F and dsNlDAP-1-R) containing a T7 polymerase promoter was designed to amplify the DNA template of NlDAP-1 dsRNA, which was 245 bp (Table 1). The dsRNA of the *GFP* gene (ds*GFP*) was used as the negative control. The *GFP* gene sequence was synthesized in vitro, referring to the binary vector pCAMBIA-1302 (GenBank: AF234298.1), and cloned into the pMD19-T vector (Takara). The *GFP* dsRNA for RNAi was 396 bp (Table 1). dsRNA was synthesized using the MEGAscript^TM^ T7 Transcription Kit (Ambion, Austin, TX, USA) according to the manufacturer’s instructions. The quality of the dsRNA products was verified via 1% agarose gel electrophoresis and the NanoDrop 2000 spectrophotometer (Thermo Scientific, Waltham, MA, USA). Approximately 50 nL of dsRNA (5000 ng/μL) was injected into the abdomen of each newly emerged virgin macropterous female using a manual microinjector.

### 2.7. Dissection Observations and Fertility Analysis

The *N. lugens* ovaries were dissected and photographed under a Nikon SMZ1500 stereomicroscope (Nikon, Tokyo, Japan) using NIS-Elements D 3.10 (Build 578) (Nikon, Tokyo, Japan). To analyze fertility, one female adult *N. lugens* injected with dsRNA (24 h after the dsRNA treatment) was mated with two untreated males in a plastic bottle (Ø: 10 cm, H: 20 cm) containing fresh rice seedlings, which were changed daily. Mortality was calculated every day, and the number of eggs laid was counted until all female *N. lugens* adults died. Three replicates were performed.

### 2.8. Statistics on the Number of Yeast-Like Symbiont in Oocytes

Mature oocytes were dissected from *N. lugens* ovaries and observed under a Nikon SMZ1500 stereomicroscope. Then, the mature oocytes were treated individually with 35% sodium hypochlorite solution to dissolve the shell and release the YLSs from the symbiont ball that was at the posterior of the mature oocyte. After all the YLSs were dispersed, they were photographed, and the number of YLSs was counted.

### 2.9. Data Analysis

Statistical analysis was conducted using one-way ANOVA followed by Tukey’s test for multiple comparisons and unpaired two-tailed Student’s *t*-tests using GraphPad Prism Software 8.0.2 (GraphPad Software, San Diego, CA, USA). All data are presented as the mean ± standard error.

## 3. Results

### 3.1. Identification and Phylogenetic Analysis of NlDAP-1

**Figure 1:**
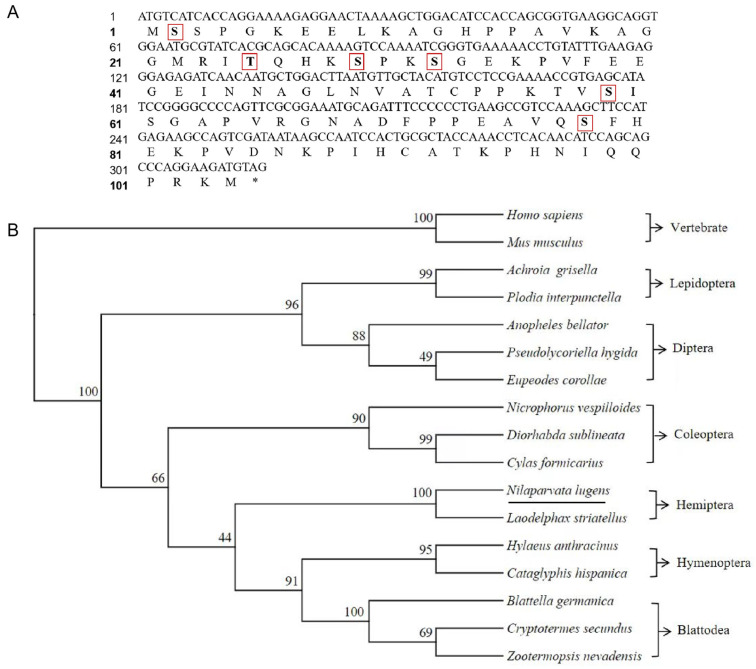
The NlDAP-1 CDS sequence (**A**) and the DAP-1 phylogenetic tree (**B**). Predicted phosphorylation modification sites are indicated by bold black font and red boxes, and termination codons are denoted by “*”. The phylogenetic tree included 17 protein sequences from *Homo sapiens* (NP_001278892.1), *Mus musculus* (NP_666169.1), *Laodelphax streatellus* (RZF42083.1), *Blattella germanica* (PSN36392.1), *Cryptotermes secundus* (XP_023707582.1), *Zootermopsis nevadensis* (XP_021925030.1), *Hylaeus anthracinus* (XP_054003797.1), *Cataglyphis hispanica* (XP_050466369.1), *Diorhabda sublineata* (XP_056633346.1), *Nicrophorus vespilloides* (XP_017781627.1), *Cylas formicarius* (XP_060536675.1), *Pseudolycoriella hygida* (KAJ6637560.1), *Eupeodes corollae* (XP_055906778.1), *Anopheles bellator* (XP_058065094.1), *Achroia grisella* (XP_059055975.1), and *Plodia interpunctella* (XP_053617949.1).

The NlDAP-1 open reading frame comprised 315 nucleotides encoding 104 amino acids (GenBank: PP836282). The *Nl*DAP-1-predicted protein was deduced with a molecular mass of approximately 11.1 kDa and a calculated pI value of 9.26. NlDAP-1 lacked a signal peptide but had a conserved domain (DAP). *Nl*DAP-1 had six phosphorylation sites, including five serine sites (S) and one threonine site (T) (Figure 1A). Sequence comparison and phylogenetic analysis showed that DAP-1 was conserved in insects, and NlDAP-1 was most similar to DAP-1 of *Laodelphax streatellus*, which is also in Hemiptera (Figure 1B).

### 3.2. Developmental and Tissue-Specific Expression of NlDAP-1

**Figure 2:**
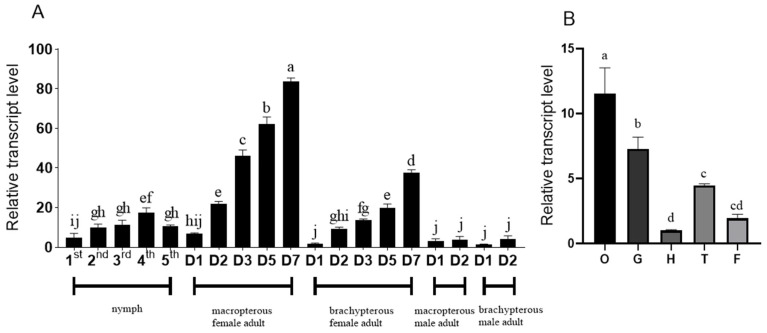
Expression of NlDAP-1 during different developmental stages and in different tissues: (**A**) NlDAP-1 expression patterns during differential developmental stages, including first to fifth nymphs, macropterous female adults (1–7 day-old), brachypterous female adults (1–7 dayold), macropterous male adults (1–2 dayold), and brachypterous male adults (1–2 dayold) (“D” in (**A**) represents “day-old” adults); (**B**) NlDAP-1 expression patterns in the head (H), ovaries (O), gut (G), thorax (T), and fat bodies (F) of macropterous females. Data are presented as the mean ± standard error (n = 3). Different lower-case letters above the bars indicate significant differences at *p* < 0.05 (one-way ANOVA performed using GraphPad Prism Software 6.0).

qPCR was used to detect the developmental and tissue-specific expression of NlDAP-1 (Figure 2). NlDAP-1 was expressed during all developmental stages, and its expression in female adults, particularly macropterous female adults, was significantly higher than in nymphs and male adults (Figure 2A). NlDAP-1 expression was also detected in the head, ovary, gut, thorax, and fat body of adult females. Among these tissues, NlDAP-1 expression was highest in the ovary, followed by the gut (Figure 2B). These results suggest that NlDAP-1 plays an important role in the reproductive development of *N. lugens*.

### 3.3. Immunofluorescence Analysis of NlDAP-1 Expression

**Figure 3:**
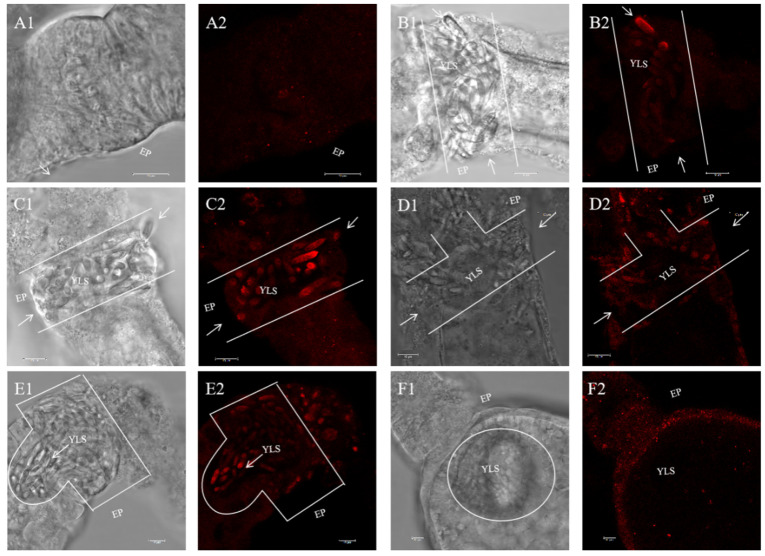
Analysis of NlDAP-1 expression using immunofluorescence: (**A1**,**A2**) YLS has not entered the epithelial plug (EP); (**B1**,**B2**) a small number of YLSs began to enter the EP; (**C1**,**C2**) a large number of YLSs entered the EP; (**D1**,**D2**) some YLSs entered the oocyte through a channel; (**E1**,**E2**) a large number of YLSs entered the oocytes and aggregated to form a symbiont ball; (**F1**,**F2**) the symbiont ball was fully formed at the posterior of the oocyte. NlDAP-1 is shown in red. The arrows in the figure indicate where YLSs enter EP. Scale bar: 10 μm (**A**–**F**).

YLSs are transovarially transmitted by entering the epithelial plug of ovariole in *N. lugens* [24]. The results show that *Nl*DAP-1 was minimally expressed at the epithelial plug (EP) of the ovarioles before the YLSs entered (Figure 3A1,A2). As the YLSs entered the ovarioles, the NlDAP-1 expression level at the EP began to rise (Figure 3B–D). When a large number of YLSs swarmed into the EP, NlDAP-1 was highly expressed at the EP (Figure 3E1,E2). When all YLSs entered the ovariole and formed a symbiont ball, the channel formed at the EP closed completely, and the NlDAP-1 expression level at the EP decreased sharply (Figure 3F1,F2). This result suggests that NlDAP-1 may be associated with the transovarial transmission of YLSs in *N. lugen*.

### 3.4. Effects of RNA Interference

**Figure 4:**
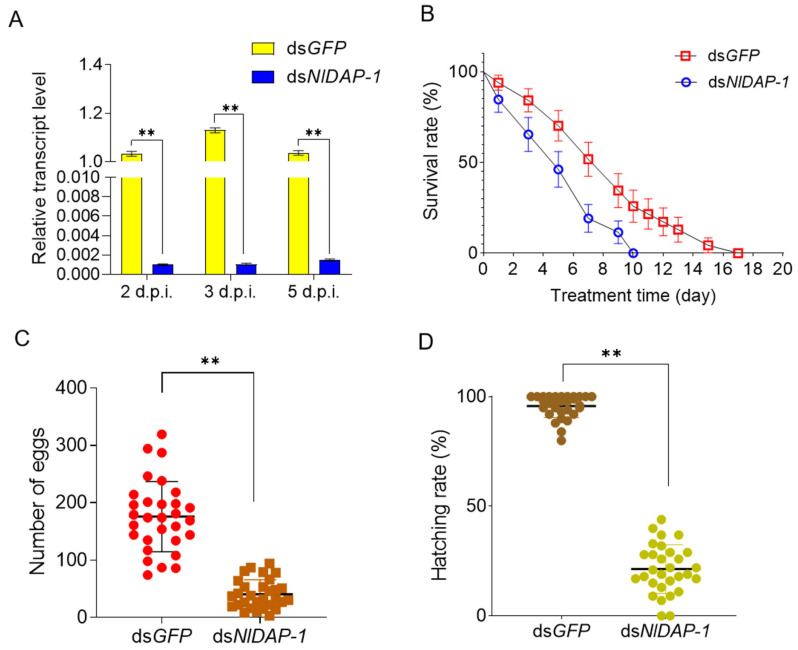
The effects of RNA interference on *N. lugens*: (**A**) downregulation of NlDAP-1 expression after the dsNlDAP-1 injection (d.p.i. represents days post dsRNA injection); (**B**) the cumulative survival rate (%) of *N. lugens* after the dsNlDAP-1 and ds*GFP* injections (50 individuals in each group); (**C**) the number of eggs laid by female adults after injecting dsNlDAP-1 (n = 30) and ds*GFP* (n = 30); (**D**) the hatching rate of eggs laid by female adults after injecting dsNlDAP-1 (n = 30) and ds*GFP* (n = 30). All data are presented as the mean ± standard error. One-way ANOVA and unpaired two-tailed Student’s *t*-tests were performed using GraphPad Prism Software 6.0. “**” in (**A**–**D**) represents *p <* 0.01.

The qPCR results demonstrated that expression of the NlDAP-1 gene in *N. lugens* was downregulated by 99.8%, 99.9%, and 99.8% at the second, third, and fifth days post NlDAP-1 dsRNA (dsNlDAP-1) injection, respectively, compared with the control group treated with ds*GFP*. This result indicates that RNAi technology effectively reduced the NlDAP-1 expression level in *N. lugens* (Figure 4A).

After the dsRNA injection, an obvious difference in survival rate was detected between the dsNlDAP-1 and ds*GFP* treatments (Figure 4B). Following the injection of dsNlDAP-1, a large number of *N. lugens* died within 3 days, and the maximum survival time of *N. lugens* was 10 days. In contrast, control *N. lugens* exhibited a maximum survival time of 17 days. The differences in survival rate between the dsNlDAP-1 and dsGFP treatment from 3 to 10 days are all significant (*p* < 0.01).

Additionally, changes in oviposition and the hatching rate of *N. lugens* were also analyzed after RNAi. After the dsNlDAP-1 injection, the average number of eggs oviposited by *N. lugens* was 41, with the highest egg count of 94 from an *N. lugens* female. This was 76.7% less than that of the ds*GFP* group, whose average number of oviposited eggs was 176 (Figure 4C). The rice seedlings used to feed *N. lugens* were dissected to count the unhatched eggs retained in the seedlings. The average number of unhatched eggs in the ds*GFP* group was only 4, with a remarkably high average hatching rate of 95.7%, while the average number of unhatched eggs in the dsNlDAP-1 group was 18, with an average hatching rate of 21.6% (77.4% reduction) (Figure 4D). These results indicate that injecting dsNlDAP-1 affects the oviposition and egg-hatching rate of *N. lugens*.

**Figure 5:**
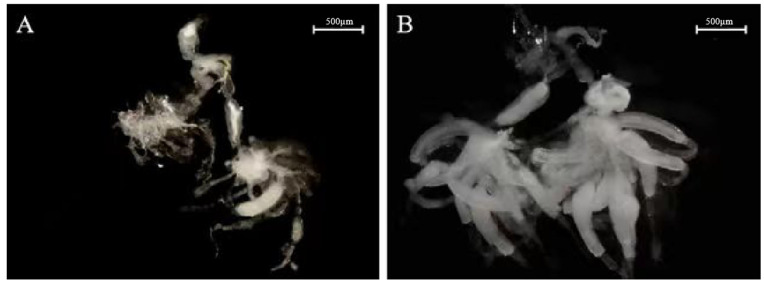
Effect of dsNlDAP-1 on *N. lugens* ovarian development: (**A**) ovaries of a female adult treated on day 3 post dsNlDAP-1 injection; (**B**) ovaries of a control female adult on day 3 post-ds*GFP* injection.

Furthermore, RNAi also affected *N. lugens* ovarian development. Up to 3 days after the dsRNA injection, a difference was detected between most ovaries from dsNlDAP-1-treated and ds*GFP*-treated females. The ovaries of females were fully developed on the third day after the ds*GFP* injection, with clearly visible banana-shaped oocytes; whereas oocytes in the ovaries of *N. lugens* treated with the dsNlDAP-1 injection were primarily immature (Figure 5A,B).

**Figure 6:**
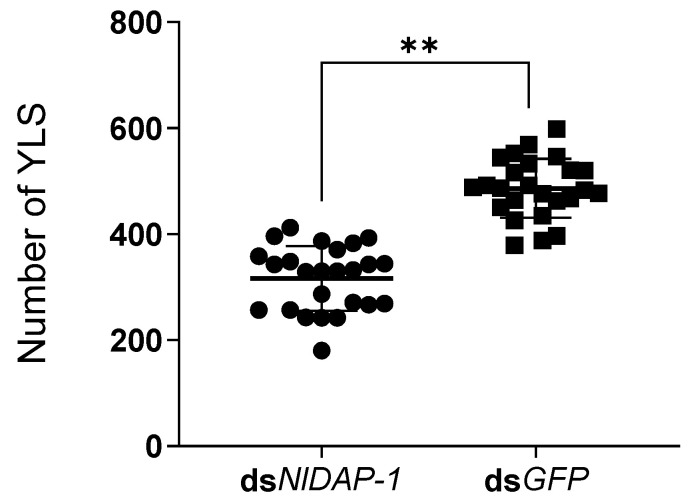
Effect of dsNlDAP-1 on the number of YLSs entering *N. lugens* oocytes. “**” indicates *p* < 0.01. Inter-group analysis and mapping were performed using the unpaired two-tailed *t*-test provided in GraphPad Prism 6.0 software.

The number of YLSs in the mature *N. lugens* oocytes on the fifth day after the dsRNA injection was recorded. The results showed that the number of YLSs in the *N. lugens* oocytes injected with dsNlDAP-1 was significantly lower than that in the ds*GFP* group, which was reduced by 34.9% (317 vs. 487 on average) (Figure 6), indicating that the entry of YLSs into *N. lugens* oocytes was disrupted by the dsNlDAP-1 injection.

## 4. Discussion

In the present study, RNAi of NlDAP-1 expression affected the survival rate, ovarian development, and egg production of *N. lugens*. These results may have occurred for the following reasons.

First, downregulation of NlDAP-1 expression may have blocked the cell development of the ovaries and then resulted in delayed ovarian development and further affected the reproductive level of *N. lugens*, because DAP-1 is involved in cell growth, migration, autophagy, and apoptosis has been verified by a large number of studies on mammalian cells [21,25]. In cancer cells, knocking down DAP-1 expression inhibits their growth and increases cell migration [26,27]. In MCF7 cells, downregulating DAP-1 also leads to decreased expression of p21 in MCF7 cells, which plays an important role in cell cycle arrest and cell senescence [18]. In SKOV3 cells, silencing DAP-1 promotes SKOV3 cell autophagy and enhances the anti-proliferation level of cardamonin to reduce cell proliferation. Taken with the high expression of NlDAP-1 in *N. lugens* ovaries, it is reasonable that RNAi of NlDAP-1 expression decreased the fecundity of *N. lugens.* This result is similar to those of other studies that are about genes highly expressed in *N. lugens* ovaries, such as *NlBRM, NlRPL5*, and *NlGro. NlBRM* is abundantly expressed in eggs and ovaries. Downregulation of *NlBRM* expression via dsRNA injection declines the oviposition of *N. lugens* females by 61.11–73.33% during the subsequent 5 days after dsRNA injection and decreases the number of newly hatched nymphs by 93.56–100% [28]. *NlRPL5* is also highly expressed in the ovaries of gravid females. RNAi of *NlRPL5* expression significantly restricts the ovarian development and decreases the number of eggs laid by *N. lugens* [29]. Similarly, after the expression of *NlGro* genes was downregulated by RNAi, the ovarian development is significantly delayed, and the number of eggs laid is reduced by 85% [30].

Second, downregulation of NlDAP-1 expression may affect the survival of hemocytes or fat body cells and, consequently, reduce the supply of some nutrient to the ovaries of *N. lugens*. Insect hemocytes are involved not only in cellular immunity but also in the producing vitellogenin and the synthesis and transport of lipoproteins [31]. A study on the function of DAP-1 in *Marsupenaeus japonicas* reported that DAP-1 enhanced the white spot syndrome virus (WSSV) infection-induced apoptosis of hemocytes, and DAP-1 knockdown decreased the virus copy number in *M. japonicus* and the mortality of *M. japonicus* with respect to WSSV challenge [25]. This means that DAP-1 affects the apoptosis of hemocytes in invertebrates. The fat body of insects is a dynamic tissue involved in multiple metabolic functions, providing essential nutrition for ovary development [32]. Whether DAP-1 is associated with the apoptosis of fat body cells in invertebrates or not has not been reported. In the present study, NlDAP-1 was found expressed in the fat body, even though the expression level was less than in the ovaries and guts. Thus, it is meaningful to study the relation between DAP-1 and fat bodies in the future.

Another interesting result in the present study is that RNAi with dsNlDAP-1 significantly decreased the number of YLSs in the oocytes of *N. lugens* and the hatching rate of eggs. The nutrients in the rice phloem are imbalanced in *N. lugens* because they lack essential amino acids, nitrogen, and sterols; thus, *N. lugens* requires symbionts, such as YLSs, to provide these essential nutrient supplements for survival and normal growth and development [33,34,35,36]. Artificially reducing the number of YLSs in *N. lugens* leads to slow growth, loss of reproduction, and premature death [37,38]. In our previous study, we reduced the number of YLSs in *N. lugens* using fungicides, such as zhongshengmycin and pymetrozine, and showed that the *N. lugens* mortality rate increased significantly and egg production by female adult *N. lugens* decreased significantly after fungicide treatment [39]. In addition, we inhibited *NlSLC26A10* and other target genes, which are crucial for YLSs to enter the ovaries, using RNAi technology and discovered that a decrease in the number of YLSs in the mature oocytes of *N. lugens* led to delayed ovarian development and decreased reproduction [40]. Therefore, the decrease in the number of YLSs in mature oocytes may have caused the reduced egg hatching rate. These results are similar to what was observed in the present study. Moreover, the immunofluorescence results suggest that NlDAP-1 was associated with YLSs entering the *N. lugens* ovary. Combining the above results, we speculate that the downregulation of NlDAP-1 expression blocked the transovarial transmission of YLSs in *N. lugens*, thereby inhibiting the egg-hatching rate.

Moreover, future attention could be paid to the regulation of the NlDAP-1 function. Since NlDAP-1 has six phosphorylation sites, the function of NlDAP-1 could be affected by phosphorylation. In some mammalian cells, dephosphorylated DAP-1 is an autophagy suppressor that inhibits cell autophagy under nutrient deprivation conditions [41,42].

## 5. Conclusions

In summary, the expression NlDAP-1 was particularly high in the ovaries of female adults, and the RNAi of NlDAP-1 caused premature death, delayed ovarian development, and blocked ovulation in *N. lugens*, as well as disturbing the transovarial transmission of YLSs, indicating that NlDAP-1 plays a crucial role in the reproductive development of *N. lugens* and the transovarial transmission of YLSs. This is the first report of a functional study of DAP-1 in insects, providing important insight into the function of DAP-1 and making DAP-1 a potential target for RNAi to control *N. lugens*. To explore the function of DAP-1 in *N. lugens* in detail, we will focus on the interaction between DAP-1 and the proteins involved in the cell apoptosis signaling pathway of *N. lugens,* as well as the interactive mechanism of DAP-1 and YLS enolase during the transovarial transmission of YLSs in *N. lugens*.

## Figures and Tables

**Table 1:** The primers used in this study.

Primers	Primer Sequence (5′-3′)
for cloning cDNA	
NlDAP-1-F	TCAGAGGAGATAAACATCGTGG
NlDAP-1-R	CTGTTGATTTCACTTTTGCTGT
for qRT-PCR	
NlDAP-1-qF	ACCAGGAAAAGAGGAACTAAAAGC
NlDAP-1-qR	CATGTAGCATTAAGTCCAGCAT
*Nl18S*-qF	GTAACCCGCTGAACCTCC
*Nl18S*-qR	GTCCGAAGACCTCACTAAATCA
for synthesizing dsRNA	
dsNlDAP-1-F	GGATCCTAATACGACTCACTATAGGGAACTAAAA
	GCTGGA
dsNlDAP-1-R	GGATCCTAATACGACTCACTATAGGCTTCTCATGG
	AAGCT
ds*GFP*-F	GGATCCTAATACGACTCACTATAGGGATACGTGCA
	GGAGAGGAC
ds*GFP*-R	GGATCCTAATACGACTCACTATAGGGCAGATTGTG
	TGGACAGG

## Data Availability

No new data were created or analyzed in this study.

## Supplementary Materials

The following supporting information can be downloaded at https://www.mdpi.com/article/10.3390/insects15060425/s1: Figure S1: The specificity of anti-DAP-1 antibody was verified by Western blot.
