# Supplementary material for: Death-Associated Protein-1 Plays a Role in the Reproductive Development of Nilaparvata lugens and the Transovarial Transmission of Its Yeast-Like Symbiont
Source: Insects. 2024 Jun 5;15(6):425. doi: 10.3390/insects15060425 (PMC11204009; doi:10.3390/insects15060425)
Supplement: Supplementary file 1 [file insects-15-00425-s001.zip › insects-2974876-supplementary.pdf]

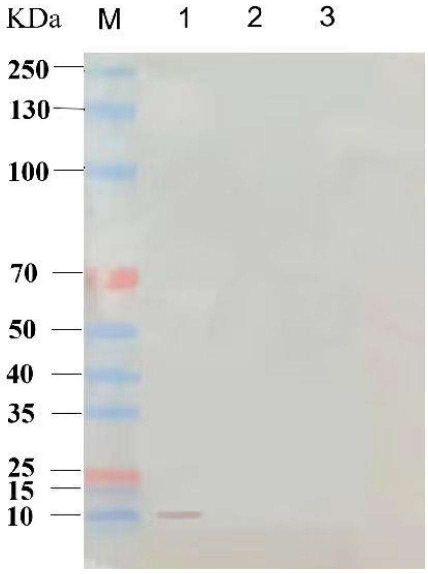

Figure S1. The specificity of anti-DAP-1 antibody was verified by western blot. The protein samples were extracted from the whole bodies of five *Nilaparvata lugens* female adults. 1. Both the primary antibody (mouse monoclonal antibody against the human DAP-1) and secondary antibody (goat polyclonal antibody against mouse IgG conjugated with HRP) were added. 2. Only the primary antibody was added. 3. Only the secondary antibody was added.
